# Supplementary material for: Recombinant human adenovirus p53 combined with transcatheter arterial chemoembolization for liver cancer: A meta-analysis
Source: PLoS One. 2023 Dec 21;18(12):e0295323. doi: 10.1371/journal.pone.0295323 (PMC10735047; doi:10.1371/journal.pone.0295323)
Supplement: S1 Appendix — (DOCX) [file pone.0295323.s001.docx]

**S5 Appendix**

| PubMed Search History | | |
| --- | --- | --- |
| #1 | Search: **"Liver Neoplasms"[Mesh]** | 186,382 |
| #2 | Search: **((((((((((((((((((((Neoplasms, Hepatic[Title/Abstract]) OR (Neoplasms, Liver[Title/Abstract])) OR (Liver Neoplasm[Title/Abstract])) OR (Neoplasm, Liver[Title/Abstract])) OR (Hepatic Neoplasms[Title/Abstract])) OR (Hepatic Neoplasm[Title/Abstract])) OR (Neoplasm, Hepatic[Title/Abstract])) OR (Cancer of Liver[Title/Abstract])) OR (Hepatocellular Cancer[Title/Abstract])) OR (Cancers, Hepatocellular[Title/Abstract])) OR (Hepatocellular Cancers[Title/Abstract])) OR (Hepatic Cancer[Title/Abstract])) OR (Cancer, Hepatic[Title/Abstract])) OR (Cancers, Hepatic[Title/Abstract])) OR (Hepatic Cancers[Title/Abstract])) OR (Liver Cancer[Title/Abstract])) OR (Cancer, Liver[Title/Abstract])) OR (Cancers, Liver[Title/Abstract])) OR (Liver Cancers[Title/Abstract])) OR (Cancer of the Liver[Title/Abstract])) OR (Cancer, Hepatocellular[Title/Abstract])** | 35,691 |
| #3 | Search: **("Liver Neoplasms"[Mesh]) OR (((((((((((((((((((((Neoplasms, Hepatic[Title/Abstract]) OR (Neoplasms, Liver[Title/Abstract])) OR (Liver Neoplasm[Title/Abstract])) OR (Neoplasm, Liver[Title/Abstract])) OR (Hepatic Neoplasms[Title/Abstract])) OR (Hepatic Neoplasm[Title/Abstract])) OR (Neoplasm, Hepatic[Title/Abstract])) OR (Cancer of Liver[Title/Abstract])) OR (Hepatocellular Cancer[Title/Abstract])) OR (Cancers, Hepatocellular[Title/Abstract])) OR (Hepatocellular Cancers[Title/Abstract])) OR (Hepatic Cancer[Title/Abstract])) OR (Cancer, Hepatic[Title/Abstract])) OR (Cancers, Hepatic[Title/Abstract])) OR (Hepatic Cancers[Title/Abstract])) OR (Liver Cancer[Title/Abstract])) OR (Cancer, Liver[Title/Abstract])) OR (Cancers, Liver[Title/Abstract])) OR (Liver Cancers[Title/Abstract])) OR (Cancer of the Liver[Title/Abstract])) OR (Cancer, Hepatocellular[Title/Abstract]))** | 200,314 |
| #4 | Search: **((Recombinant human adenovirus-p53[Title/Abstract]) OR (rAd-p53[Title/Abstract])) OR (Gendicine[Title/Abstract])** | 130 |
| #5 | Search: **(("Liver Neoplasms"[Mesh]) OR (((((((((((((((((((((Neoplasms, Hepatic[Title/Abstract]) OR (Neoplasms, Liver[Title/Abstract])) OR (Liver Neoplasm[Title/Abstract])) OR (Neoplasm, Liver[Title/Abstract])) OR (Hepatic Neoplasms[Title/Abstract])) OR (Hepatic Neoplasm[Title/Abstract])) OR (Neoplasm, Hepatic[Title/Abstract])) OR (Cancer of Liver[Title/Abstract])) OR (Hepatocellular Cancer[Title/Abstract])) OR (Cancers, Hepatocellular[Title/Abstract])) OR (Hepatocellular Cancers[Title/Abstract])) OR (Hepatic Cancer[Title/Abstract])) OR (Cancer, Hepatic[Title/Abstract])) OR (Cancers, Hepatic[Title/Abstract])) OR (Hepatic Cancers[Title/Abstract])) OR (Liver Cancer[Title/Abstract])) OR (Cancer, Liver[Title/Abstract])) OR (Cancers, Liver[Title/Abstract])) OR (Liver Cancers[Title/Abstract])) OR (Cancer of the Liver[Title/Abstract])) OR (Cancer, Hepatocellular[Title/Abstract]))) AND (((Recombinant human adenovirus-p53[Title/Abstract]) OR (rAd-p53[Title/Abstract])) OR (Gendicine[Title/Abstract]))** | 24 |
