# Supplementary figures and images for: Recombinant human adenovirus p53 combined with transcatheter arterial chemoembolization for liver cancer: A meta-analysis
Source: PLoS One. 2023 Dec 21;18(12):e0295323. doi: 10.1371/journal.pone.0295323 (PMC10735047; doi:10.1371/journal.pone.0295323)

**S1 Fig**

**A**

**
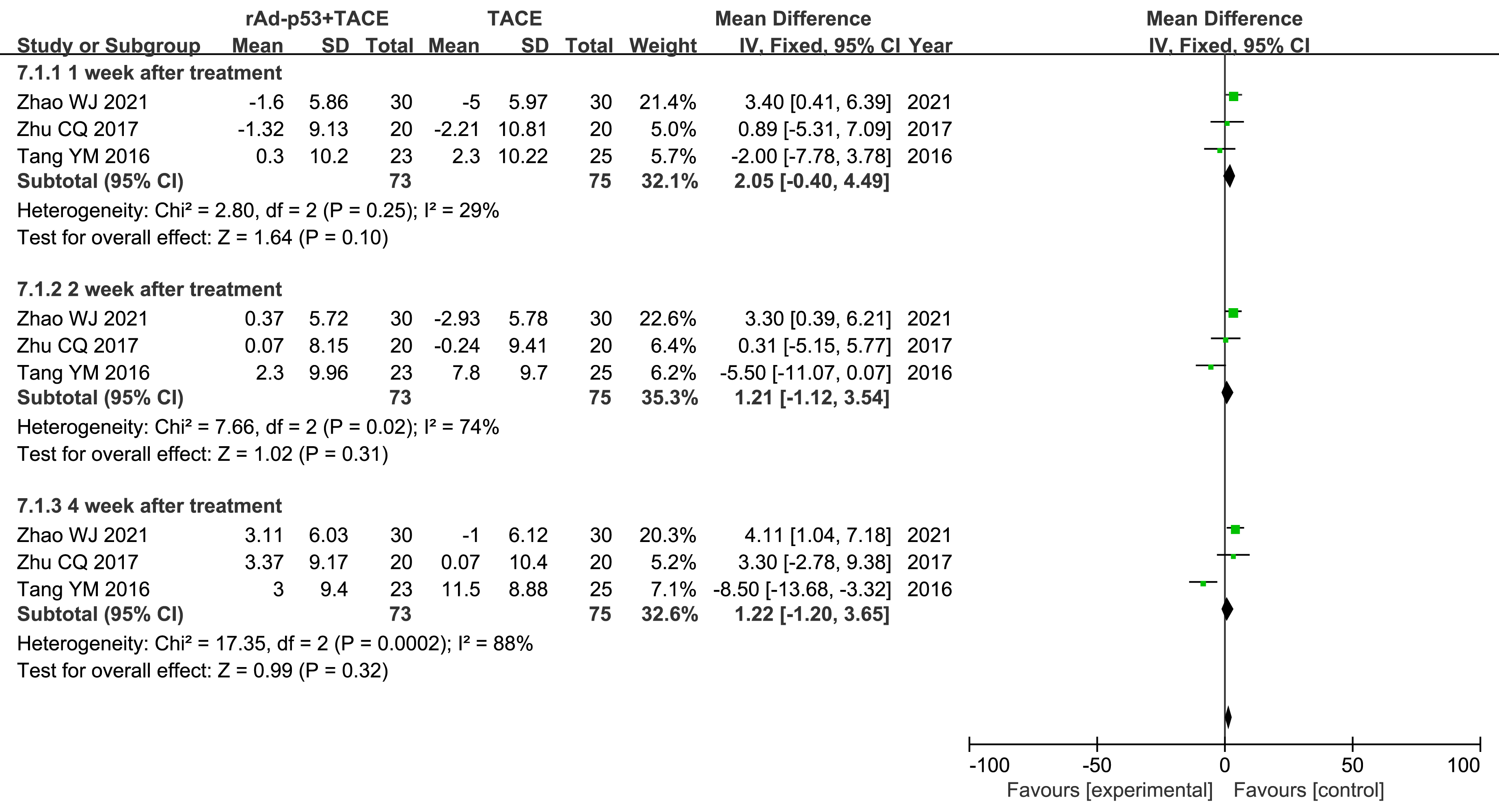
**

**B**

**
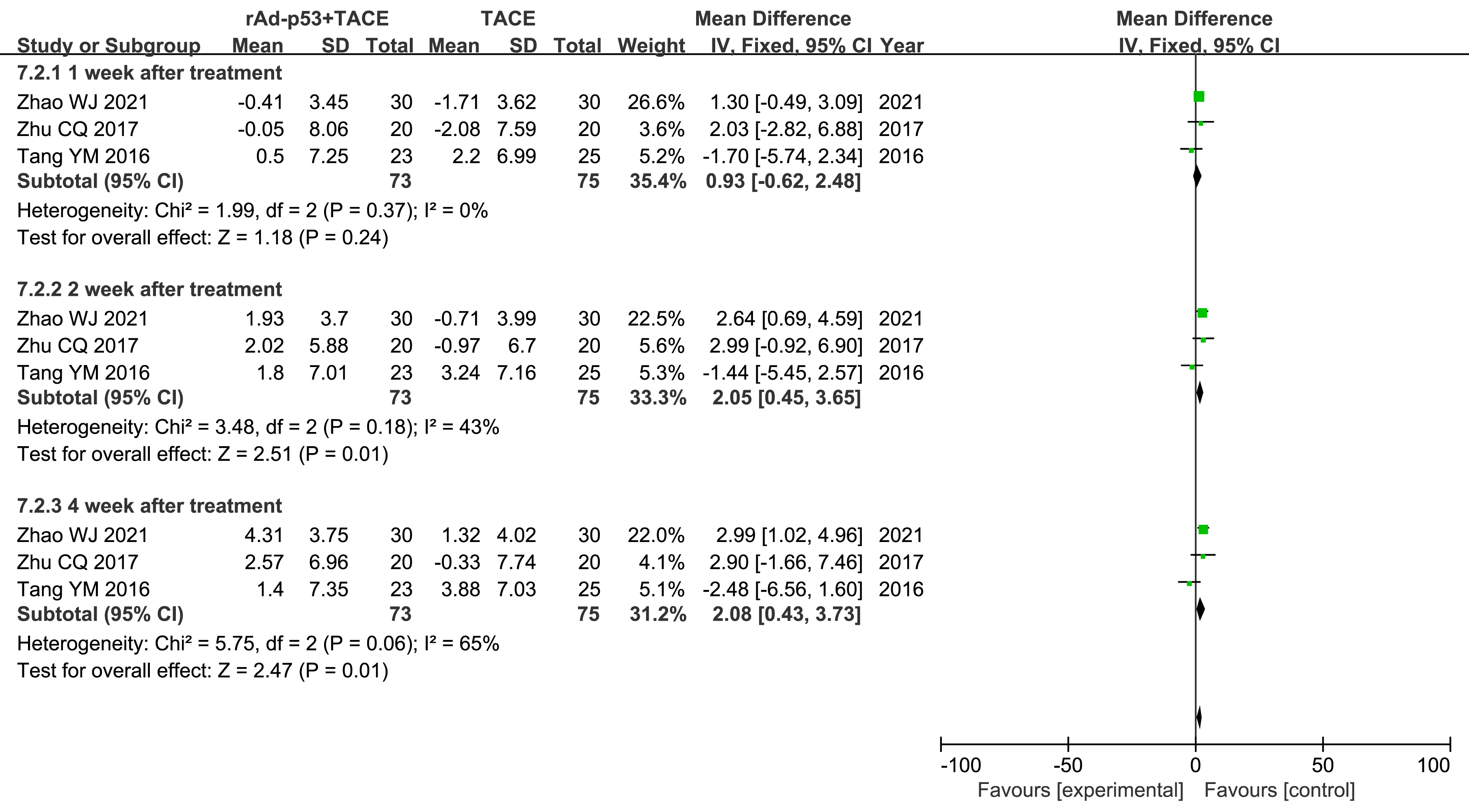
**

**C**

**
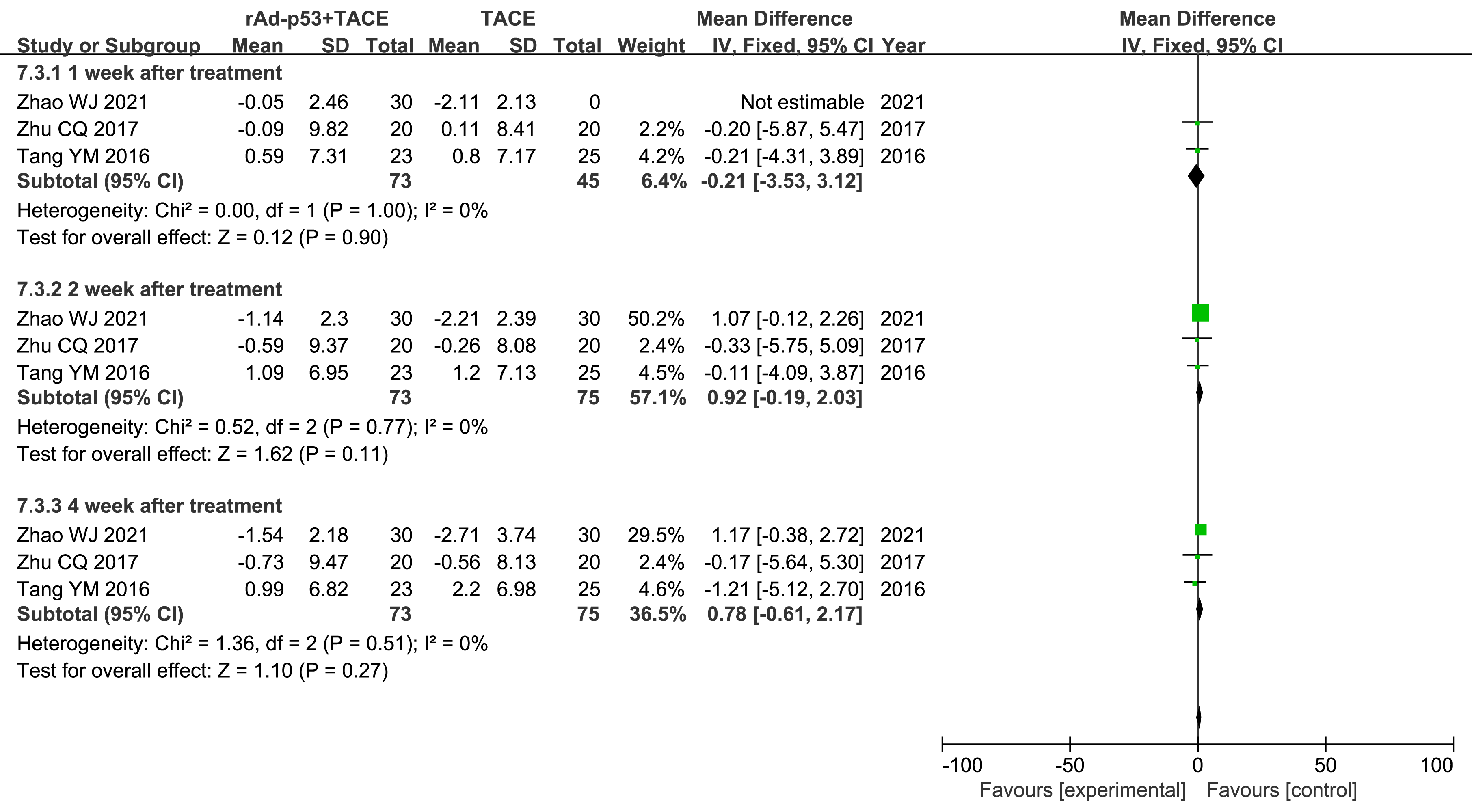
**

**D**

**
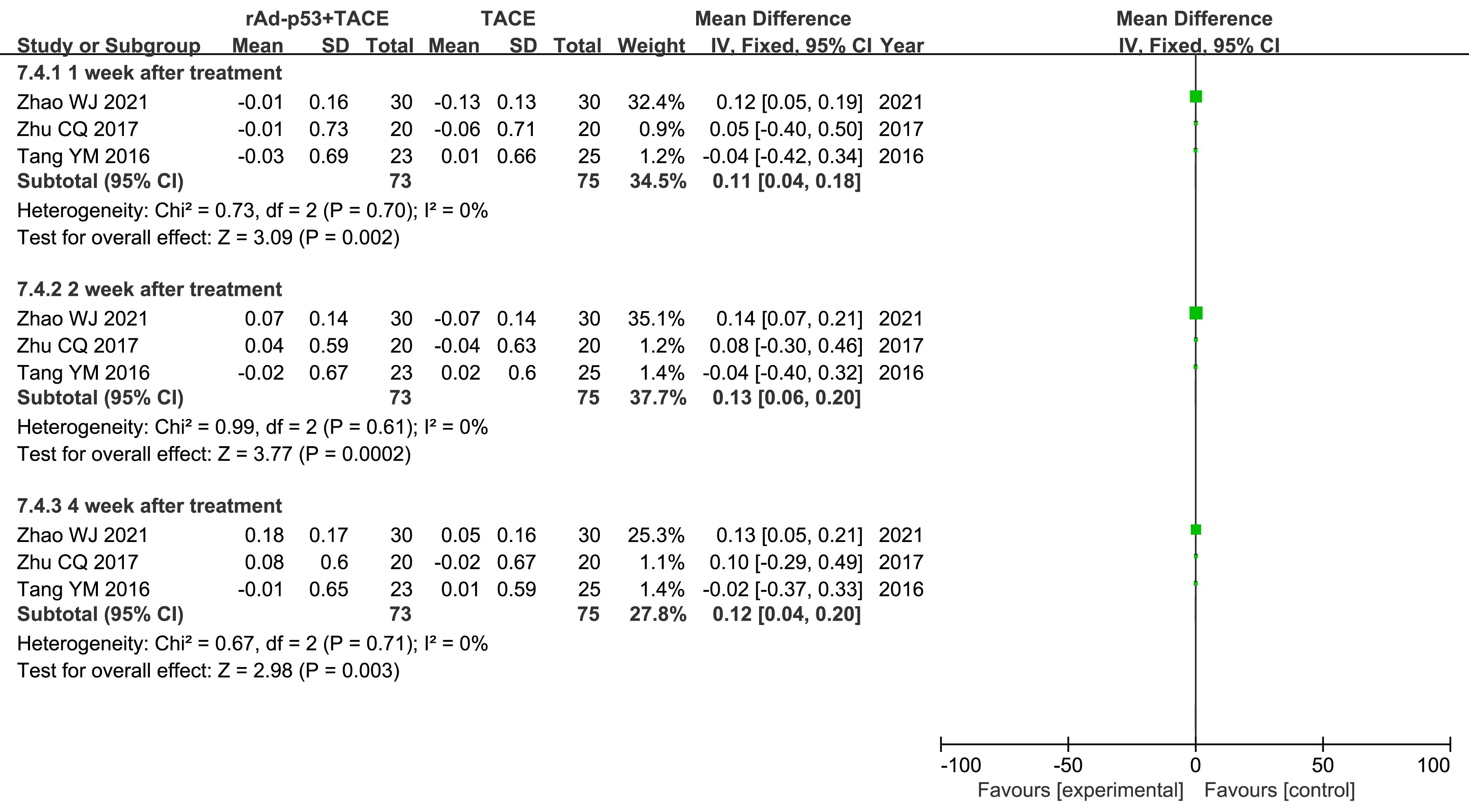
**

Supplement: S1 Fig — CD3+ (A), CD4+(B), CD8+(C), CD4+/CD8+(D). (DOCX) [file pone.0295323.s002.docx]

**S2 Fig**

**A B**


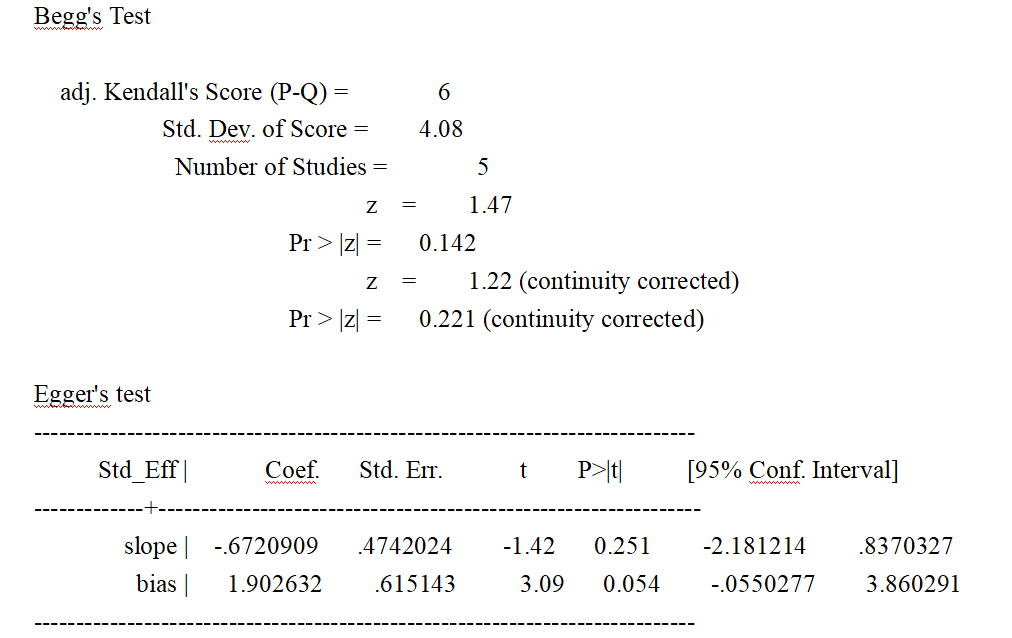

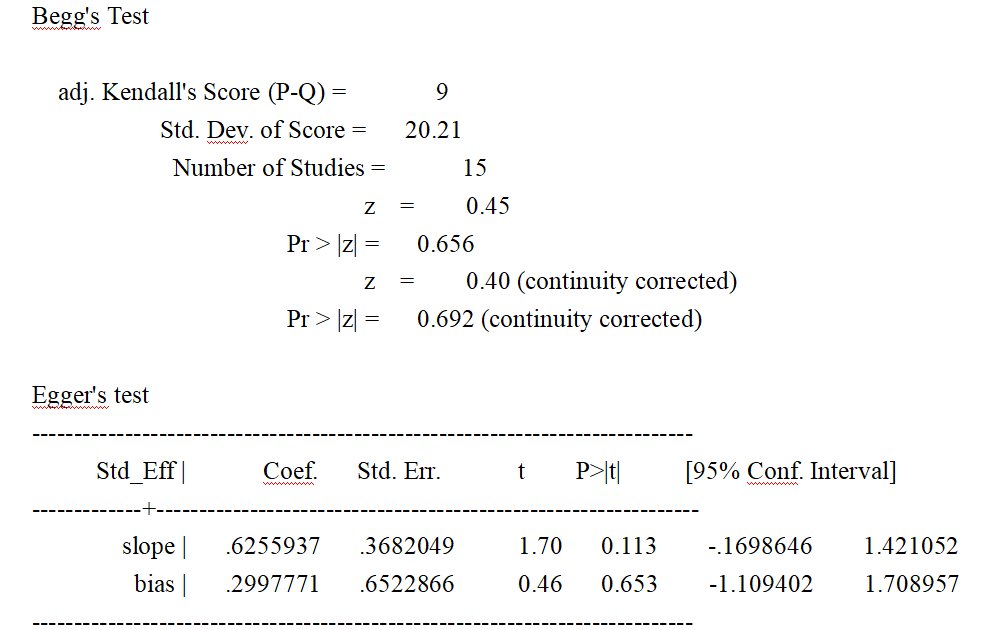


**C D**


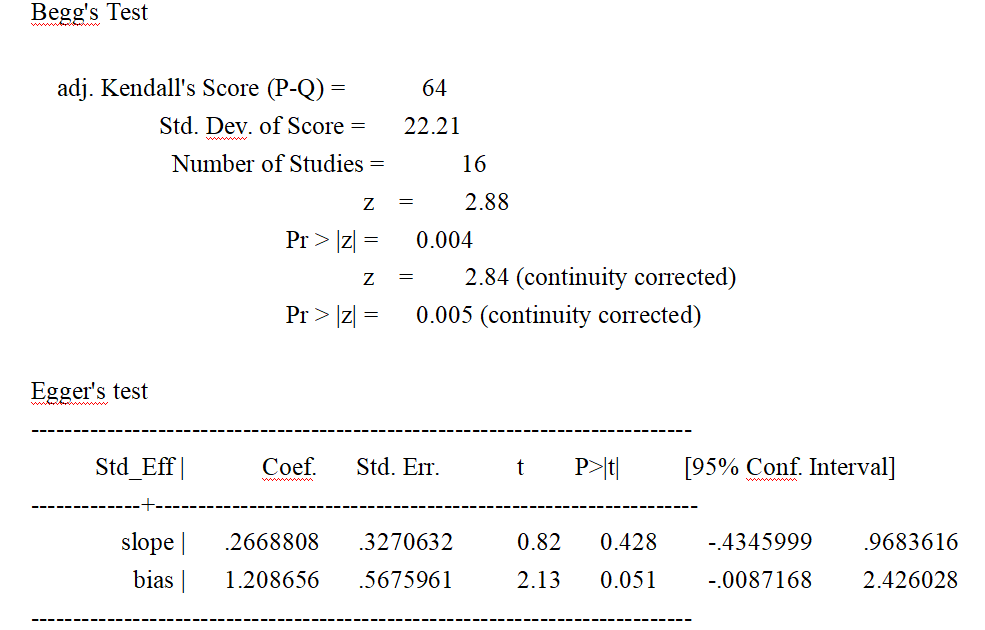

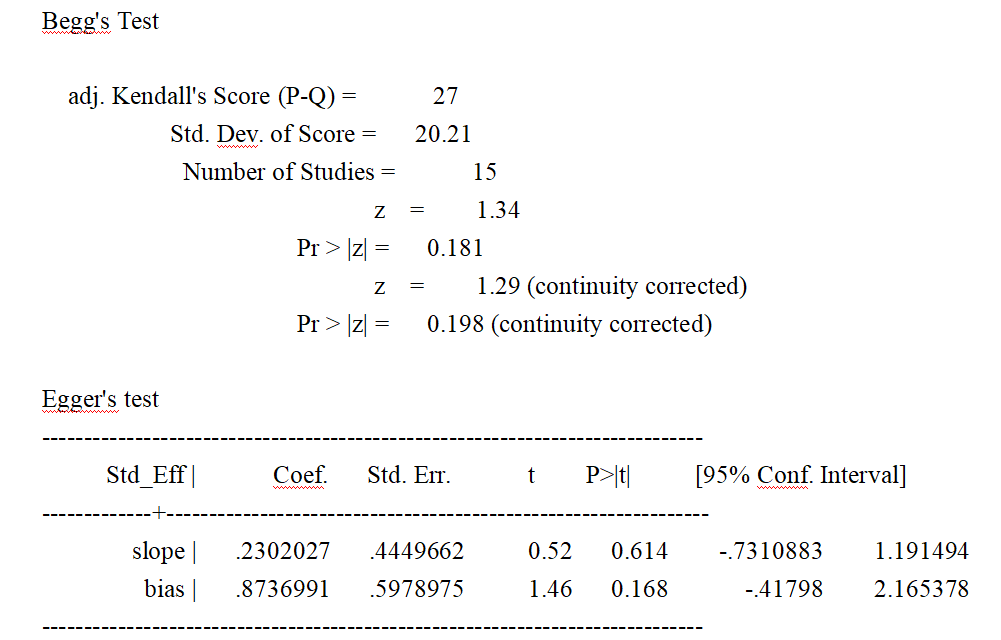

Supplement: S2 Fig — Begg’s and Egger’s tests for publication bias of CR(A), PR(B), ORR(C), DCR(D). (DOCX) [file pone.0295323.s003.docx]

**S3 Fig**

**A**

**
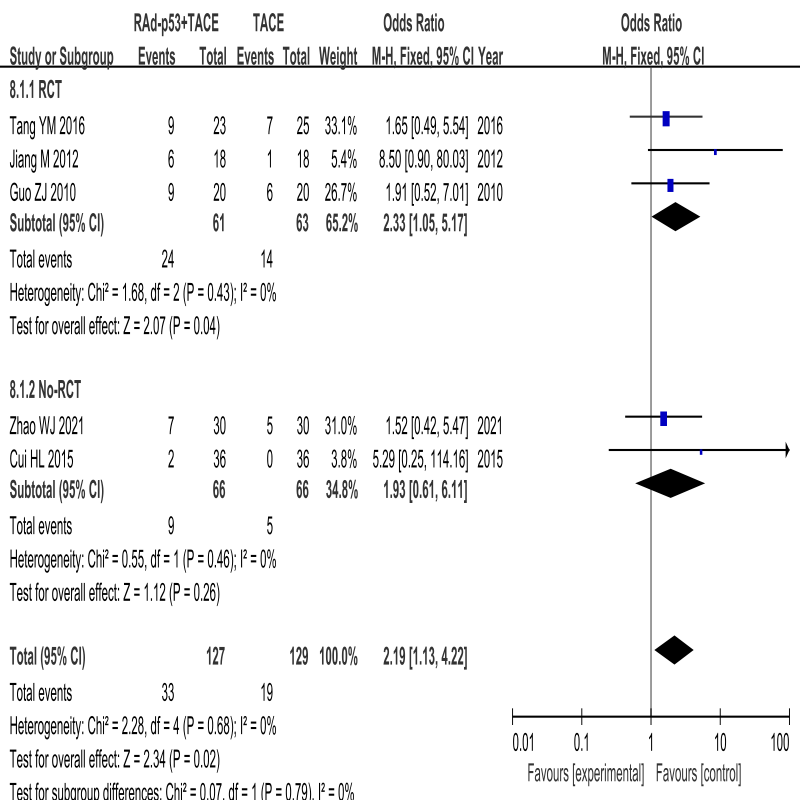
**

**B**


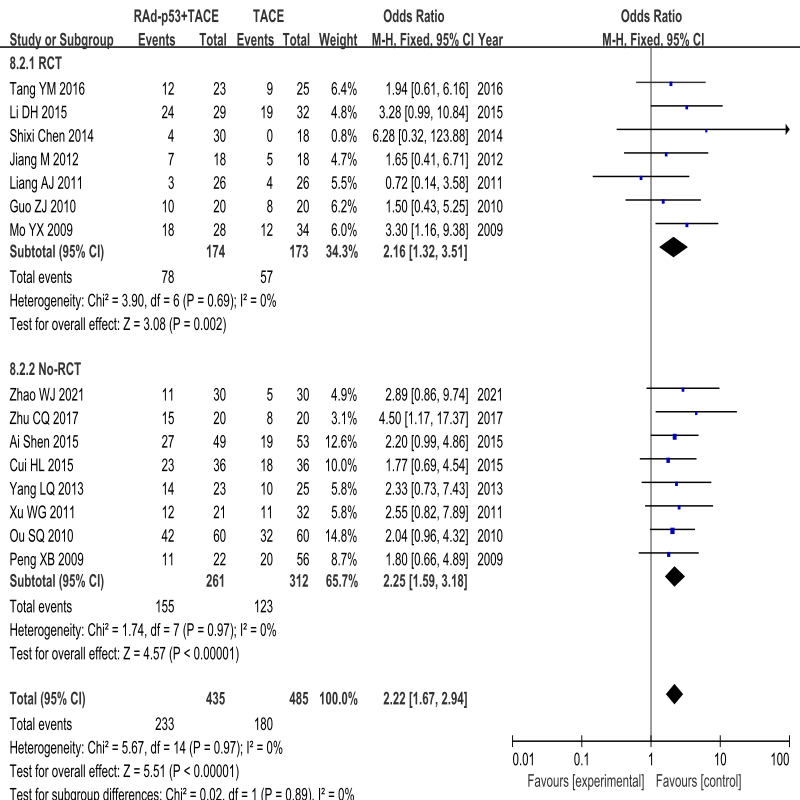


**C**


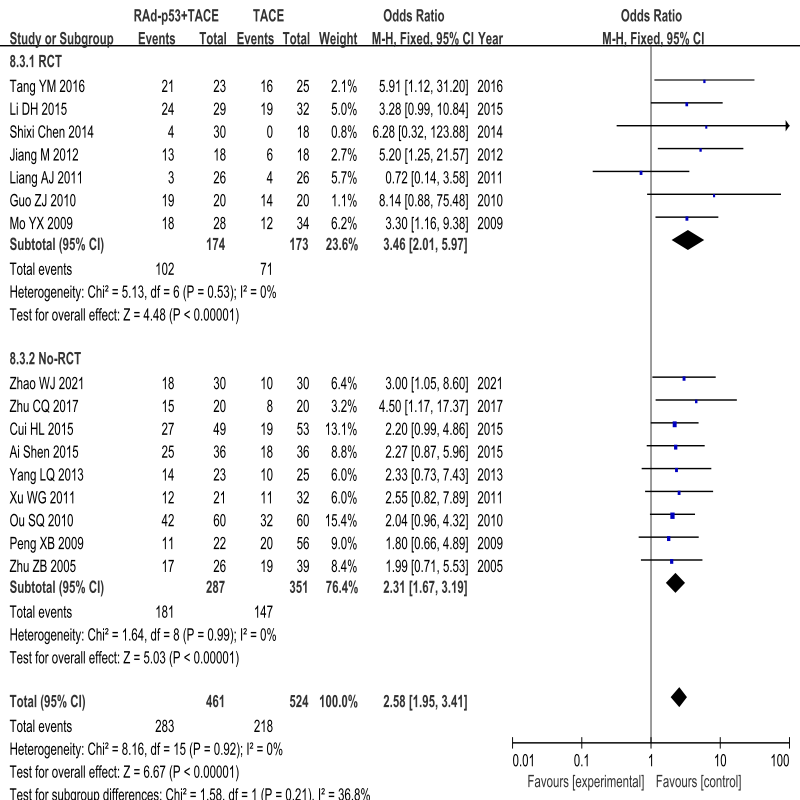


**D**


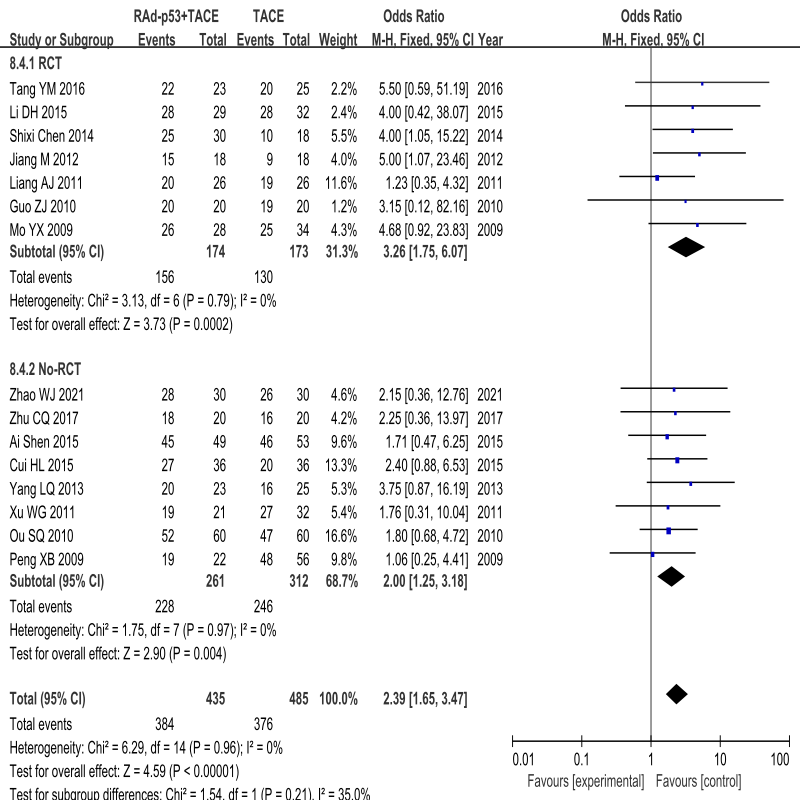

Supplement: S3 Fig — Forest plot for subgroup analysis of CR(A), PR(B), ORR(C) and DCR(D) based on RCT/Non-RCT. (DOCX) [file pone.0295323.s004.docx]
