# Supplementary material for: Recombinant human adenovirus p53 combined with transcatheter arterial chemoembolization for liver cancer: A meta-analysis
Source: PLoS One. 2023 Dec 21;18(12):e0295323. doi: 10.1371/journal.pone.0295323 (PMC10735047; doi:10.1371/journal.pone.0295323)
Supplement: Attachment — Submitted filename: Second Response to Reviewers.docx [file pone.0295323.s007.docx]

**Dear Reviewers ：**

Thank you for your letter and the reviewers’ comments concerning our manuscript entitled “

Recombinant human adenovirus p53 combined with transcatheter arterial chemoembolization for liver cancer: a meta-analysis” (PONE-D-23-04833). We really appreciate editors and reviewers greatly for their positive and constructive comments and suggestions on our manuscript. Those comments and suggestions are all valuable and very helpful for revising and improving our paper. We have read through comments carefully and have made corrections. Based on the instructions provided in your letter, we uploaded the file of the revised manuscript. Revisions in the text are shown using red highlight for additions.We would love to thank you for allowing us to resubmit a revised copy of the manuscript and the time and effort you put into our manuscripts.

**Answer to the question of reviewer four**

**Question 1：**What do you mean by primary liver tumor? HCC? Cholangio ?? both??

**My answer：**

Thank you very much for your question and I apologize for not stating that it was our problem. Primary liver cancer (PLC) is a malignant tumor that occurs in the epithelial cells of hepatocytes or intrahepatic bile ducts. Primary liver cancer mainly includes three pathological types: hepatocellular carcinoma (HCC), intrahepatic cholangiocarcinoma (ICC), and combined hepatocellular-cholangiocarcinoma (cHCC-CCA). Among them, hepatocellular carcinoma (HCC) accounts for about 85-90% of all primary liver malignancies.

Cholangiocarcinoma (CCA) is a malignant tumor originating from the extrahepatic bile duct.According to their location, they are classified as carcinoma of the perihilar bile ducts,carcinoma of the hilar bile ducts and carcinoma of distal extrahepatic bile ducts.

We re-examined the 17 studies we included and found that 11 papers included patients with primary liver cancer and another six included patients with hepatocellular hepatocellular carcinoma, and given that hepatocellular hepatocellular carcinoma makes up a major portion of primary liver cancers, we believe that the inclusion of patients with hepatocellular hepatocellular carcinoma was almost exclusively in this meta-analysis.

**Question 2:** What is the authors explanation of higher GI symptoms and myelosuppression in TACE only group ?

**My answer：**

We investigated the cause of side effects in the two groups because we wanted to prove that the addition of rAd-p53 in the experimental group did not increase the original side effects of TACE, and to prove the safety of Rad-p53. the results of meta-analysis proved that the incidence of side effects in the experimental group was lower than that in the control group. Given that only Rad-p53 was different between the experimental group and the control group, we believe that Rad-p53 may have altered the immune response of the patients in the experimental group, leading to a reduction in side effects, which is also a point of great interest to us, and we will further study the alteration of the immune environment of the patients by Rad-p53 in the future. In addition, given that the patients in the experimental group all signed the informed consent for Rad-p53, we think that there may be some psychological implication for the patients in the experimental group.

**Question 3：**Discussion should be broken into smaller paragraphs. Please indent the beginning of each paragraph and justify your whole text.

**My answer：**

Thank you very much for the heads up, sorry for the error, we have revised our discussion section as you requested.
